# The care of critically ill infants and toddlers in neonatal intensive care units across Italy and Europe: our proposal for healthcare organization

**DOI:** 10.1007/s00431-021-04349-9

**Published:** 2022-01-28

**Authors:** Nicola Pozzi, Paola Cogo, Corrado Moretti, Paolo Biban, Tiziana Fedeli, Luigi Orfeo, Eloisa Gitto, Fabio Mosca

**Affiliations:** 1 Neonatal Intensive Care Unit, Department of Maternal and Child Health, San Pio Hospital, Via dell’Angelo 1, Benevento, 83013 Italy; 2 Department of Medicine (DAME), Division of Pediatrics, S. Maria della Misericordia University Hospital, University of Udine, P.zzale S. Maria della Misericordia, 15, Udine, 33100 Italy; 3 Emeritus Consultant in Paediatrics, Policlinico Umberto I, Sapienza University, Rome, Italy; 4 Department of Neonatal and Paediatric Critical Care, Verona University Hospital, Verona, Italy; 5 Neonatal Intensive Care Unit, Fondazione Monza e Brianza per il Bambino e la sua Mamma and Azienda Socio Sanitaria Territoriale-Monza, Monza, Italy; 6 Neonatal Intensive Care Unit, San Giovanni Calibita Fatebenefratelli Hospital, Rome, Italy; 7 Neonatal and Paediatric Intensive Care Unit, Department of Human Pathology in Adult and Developmental Age “Gaetano Barresi, ” University of Messina, Via Consolare Valeria, 1, Messina, 98125 Italy; 8 Neonatal Intensive Care Unit, Department of Clinical Science and Community Health, IRCCS Foundation Ca’ Granda Ospedale Maggiore Policlinico, University of Milan, Milan, Italy

**Keywords:** Management of critically ill infants and toddlers, Paediatric critical care medicine (PCCM), Neonatal intensive care units (NICUs), Paediatric intensive care units (PICUs), Early childhood intensive care units (ECICUs), Neonatologists

## Abstract

Numerous studies have shown that critically ill infants and toddlers admitted to paediatric intensive care units (PICUs) have a lower mortality than those admitted to adult ICUs. In 2014, there were only 23 registered PICUs in Italy, most of which were located in the north. For this reason, in Italy and elsewhere in Europe, some neonatal ICUs (NICUs) have begun managing critically ill infants and toddlers. Our proposal for healthcare organization is to establish “extended NICUs” in areas where paediatric intensive care beds are lacking. While some countries have opted for a strict division between neonatal and paediatric intensive care units, the model of “extended NICUs” has already been set up in Italy and in Europe. In this instance, the management of critically ill infants and toddlers undoubtedly falls upon neonatologists, who, however, must gain specific knowledge and technical skills in paediatric critical care medicine (PCCM). Postgraduate residencies in paediatrics need to include periods of specific training in neonatology and PCCM. The Italian Society of Neonatology’s Early Childhood Intensive Care Study Group is supporting certified training courses for its members involving both theory and practice.

*Conclusion*: Scientific societies should promote awareness of the issues involved in the intensive management of infants and toddlers in NICUs and the training of all health workers involved. These societies include the Italian Society of Neonatology, the European Society of Paediatric and Neonatal Intensive Care, and the Union of European Neonatal and Perinatal Societies. They should also act in concert with the governmental institutional bodies to establish the standards for the “extended NICUs.”

**Table:** 

**What is Known:** *• The mortality of critically ill infants and toddlers admitted to PICUs is lower than that for those admitted to adult ICUs.* *• In Italy, there are only a handful of PICUs, located mainly in the north.*	
**What is New:** *• Critically ill infants and small toddlers can be managed in “extended NICUs” in areas with a lack of paediatric intensive care beds.* *• “Extended NICUs” is our proposal for healthcare organization to compensate for the paucity of paediatric intensive care beds, but neonatologists must be trained to provide them with specific knowledge and technical skills in PCCM.*

**What is Known:**

*• The mortality of critically ill infants and toddlers admitted to PICUs is lower than that for those admitted to adult ICUs.*

*• In Italy, there are only a handful of PICUs, located mainly in the north.*

**What is New:**

*• Critically ill infants and small toddlers can be managed in “extended NICUs” in areas with a lack of paediatric intensive care beds.*

*• “Extended NICUs” is our proposal for healthcare organization to compensate for the paucity of paediatric intensive care beds, but neonatologists must be trained to provide them with specific knowledge and technical skills in PCCM.*

## Introduction

In recent years, a number of studies have shown that critically ill children admitted to paediatric intensive care units (PICUs) receive higher quality of care and have better outcomes and lower mortality than those admitted to adult ICUs, both in Italy [1] and in developing countries [2]. Moreover, several studies have shown that neonatal intensive care units (NICUs) and PICUs with high volume of patients exhibit a better outcome compared with ICU with low volume of paediatric admissions [3–5]. Despite this, as highlighted in a recent editorial, there is a critical paucity of PICUs in Italy as well as a significant discrepancy between north and south [6]. In 2014, the Italian Paediatric Intensive Care Units Network (TIPNet) recorded only 23 PICUs, with a total presumed number of beds of 202, serving a population of around 60 million; of these, 11 are located in the north, 8 in central Italy, and just 4 in the south and islands.

Even if it is very difficult to register the real number of paediatric intensive care beds in Italy because there is no identification code for the discipline, differently to all other branches of medicine, it is possible to hypothesize that the ratio of beds to children is also very low compared with other countries: 1:18,000 in the USA, 1:25,000 in the UK, and just 1:75,000 in Italy (North Italy 1:50,000, South Italy 1:150,000). Consequently, significant numbers of children are inappropriately admitted to adult facilities every year, where they may not receive the minimum standards of care for their disease and age.

Alongside this, the improved quality of care in NICUs in recent years has led to an increase in the survival of preterm infants and toddlers with chronic respiratory diseases such as bronchopulmonary dysplasia, severe neurological damage from perinatal asphyxia or prematurity, and children with medical complexity. As a result, the lack of paediatric intensive care beds is often exacerbated by the low turnover of beds for acute patients because beds are occupied by chronically ill long-stay patients [7].

To address the chronic lack of paediatric intensive care beds, over the years, some Italian NICUs have offered intensive care to infants and toddlers with acute (predominantly respiratory) medical conditions and to critically ill children with medical complexity; altogether, this paediatric population represents the greater part of the children who need intensive care.

In this article, we will review the Italian experience on the care of critically ill children admitted to “extended NICUs” compared to the other European countries where the children are centralized to PICUs.

## Epidemiological background in Italy

According to Italy’s Office of National Statistics (ISTAT), over the last 30 years, there has been a gradual but significant drop in both neonatal mortality (from 6.2/1000 in 1990 to 2.0/1000 in 2018) and infant mortality (from 8.3/1000 in 1990 to 3.05/1000 in 2018) [8].

A comparison with other industrialized countries finds Italy in 7th place worldwide in the ranking of countries with the lowest rate of infant mortality, demonstrating the efficacy of Italy’s social and health service and its high quality of life [9].

However, while the neonatal mortality rate in the north is just 1.5/1000, in the centre and south (including Sardinia and Sicily), this rises to around 3.0/1000. There is a similar difference in infant mortality, with values much lower in the north (around 2.5/1000) than in the rest of Italy (around 4.0/1000) [10]. Of note, several European countries exhibit an infant mortality rate very similar to that reported in the centre and south of Italy according to the recent 2018 EUROSTAT data [11].

In 75% of cases, death in the first month of life is caused by perinatal diseases, i.e. disorders arising between the last period of pregnancy and the first week of life. Reducing neonatal mortality thus requires identifying and intervening in perinatal pathological events.

By comparison, only 18% of cases of death in the first year of life are due to perinatal diseases, with the remainder caused, in decreasing order, by congenital, respiratory, cardiac and digestive diseases, etc. (ISTAT data). Reducing infant mortality thus requires the early identification and treatment of all diseases arising in the first year of life.

It can be seen from this analysis that there is a disparity in the organization of and access to healthcare, including paediatric intensive care, between the three main regions of Italy (north, centre, and south/islands), even if mortality is obviously a multifaceted and complex outcome unlikely to be significantly influenced by the only NICU/PICU setting.

Moreover, it is conceivable that the disparity in access to the healthcare system may have a greater impact on morbidity than the mortality of critically ill infants needing intensive care. Unfortunately, no data are available to elucidate this speculation.

## Distribution of PICUs in Italy

In Italy, it is difficult to accurately account for the number of PICUs and of the PICU beds because the identification code for the discipline is missing.

For this reason, we analyzed data from the Italian Network of Paediatric Intensive Care Units (TIPNet) report for 2010–2014, published on the Cineca website. TIPNet is a PICU network that records all PICU admissions on a voluntary basis. The report reveals that Italy has few PICUs in relation to its population (23 PICUs, of which 11 in the north, 8 in the centre, and just 4 in the south and islands). There is also a great discrepancy between the different regions. For example, Lombardy has around 10 million inhabitants and 4 PICUs, and thus has an adequate provision, according to Italian Permanent Conference for paediatric healthcare guidelines of 21 December 2017, which affirms that PICUs are a key part of the emergency network and should be located in a specialist paediatric hub with a user base of at least 2 million inhabitants. The same is true of Lazio, with around 6 million inhabitants and 3 PICUs. In contrast, Campania also has around 6 million inhabitants but just one PICU, which often also takes in critically ill children from its neighbours, Puglia and Calabria.

The TIPNet report also reveals that the median age of children admitted to PICUs is 19.7 months (range 0–192 months). Infants account for around 30% of all admissions. Around 55% are admitted to a PICU for medical problems, and about half of these for an acute respiratory condition. Around 45% are children with comorbidities, of which about 30% are infants.

However, in areas where PICU beds are not available, critically ill infants and toddlers are often admitted to the NICU. Thus, it is conceivable that in the TIPNet report, a significant proportion of critically ill children are missing. To better define the clinical characteristics of this subgroup of infants and toddler, the Early Childhood Intensive Care (ECICU) Study Group is setting up an on-line registry endorsed by the Italian Society of Neonatology (ISN) with the aim to obtain more precise epidemiological data and to support the Italian Ministry of Health to define the real number of PICU beds in the country.

## Organization of NICUs in Italy

**Fig. 1:**
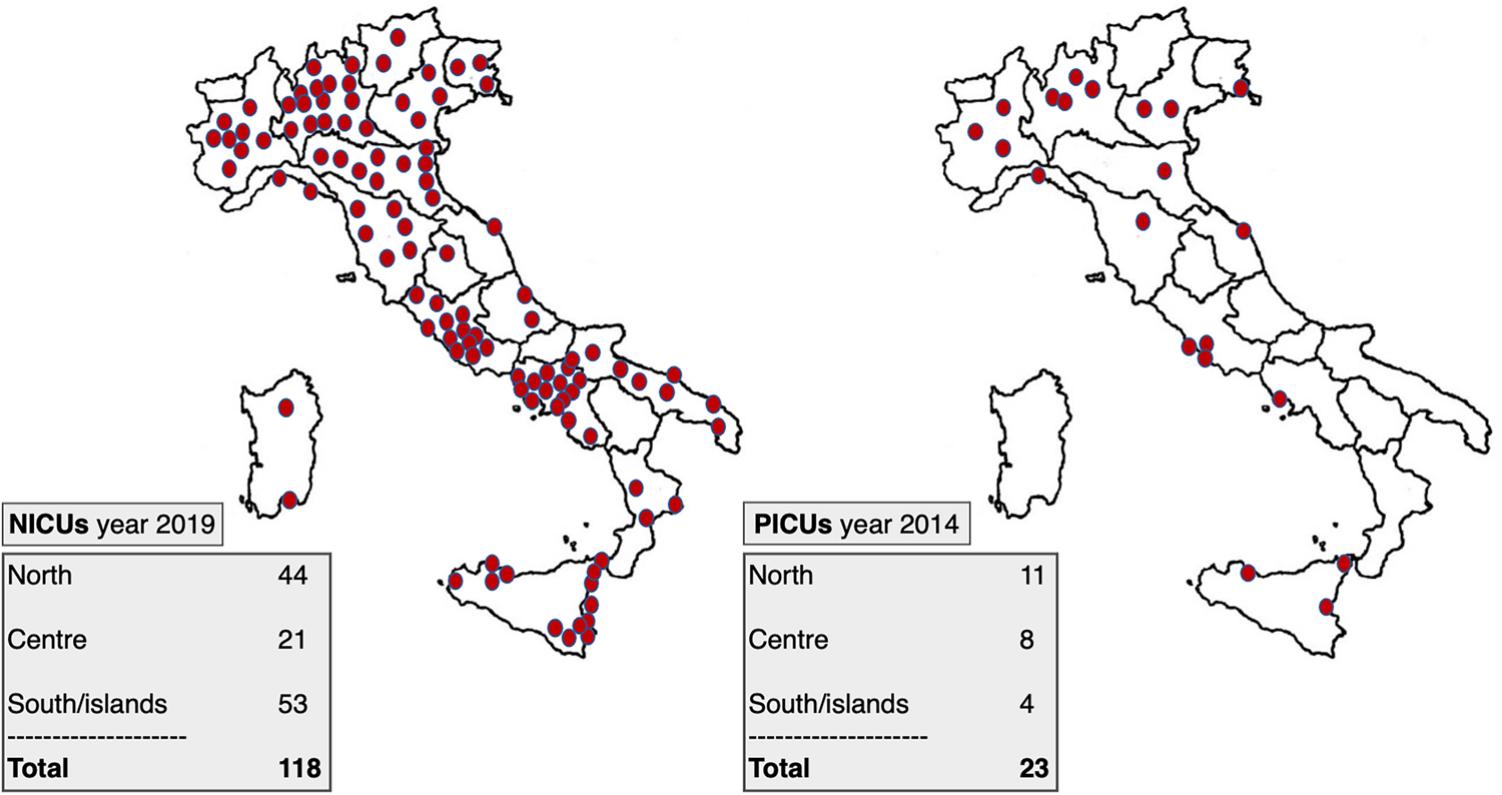
Italy: distribution of NICUs compared to PICUs

In recent years, NICUs in Italy and Europe have achieved high levels of healthcare, with a significant increase in neonatal survival. In 2019, there were 118 NICUs in Italy (data reported by the regional sections of the Italian Society of Neonatology), distributed evenly around the country (Fig. 1).

To address the chronic lack of paediatric intensive care beds, especially in areas of Italy without any PICUs, some NICUs have, over the years, offered intensive care to infants and toddlers with acute (predominantly respiratory) medical conditions and children with medical complexity, who comprise an ever-greater part of the critically ill paediatric population with conditions requiring highly complex care.

An unpublished survey of “extended NICUs” carried out by ISN in 2015 revealed that in 66% of the 92 NICUs that responded, patients aged over 28 days and/or with a post-conceptional age of 44 weeks had been admitted to neonatal facilities (in 78% of cases for respiratory diseases). Sixty five of these NICUs admitted less than 20 patients a year, 8 from 20 to 50, and 6 between 50 and 100 patients a year, revealing a significant problem in relation to the volume of paediatric admissions and the long-term maintenance of professional skills.

For this reason, one of the objectives of ISN’s ECICU Study Group is to work alongside the governmental institutional bodies to help organize and define the standards for “extended NICUs” in the areas with the greatest need for paediatric intensive care beds.

## Our proposal for healthcare organization: early childhood intensive care units ( ECICUs) or “extended NICUs”

Many of the problems experienced by infants and children in intensive care first arise in NICUs, and there is an affinity — in terms of both disease and age — with the conditions usually managed by neonatologists.

**Fig. 2:**
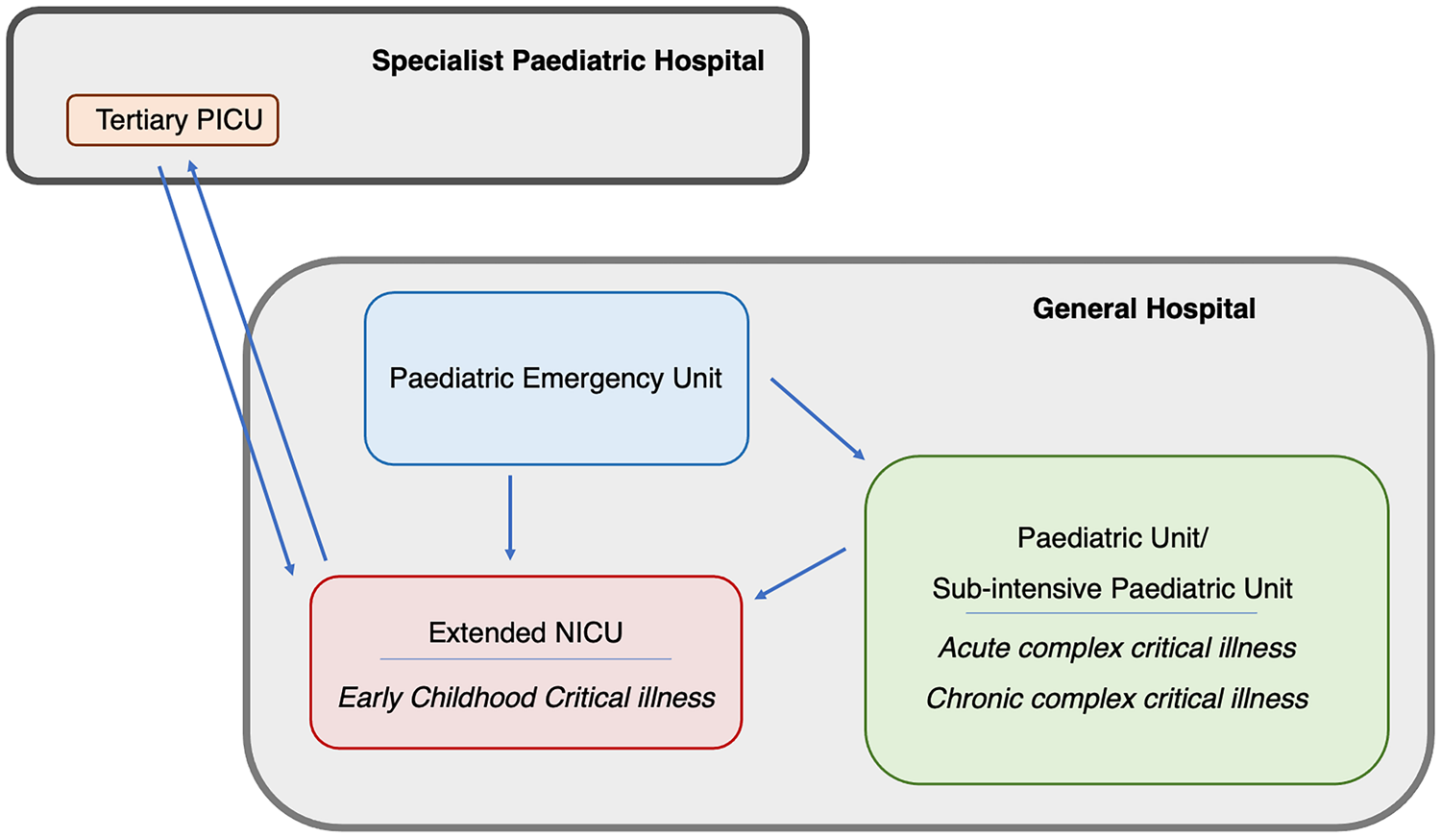
Our proposal for healthcare organization: early childhood intensive care units (ECICUs) or “extended NICUs”

Given this, our proposal for healthcare organization is the extension of some NICUs around Italy, in areas with few or even no PICU beds, to cover infants and toddlers for some specific diseases. We have called these new care facilities ECICUs or “extended NICUs” (Fig. 2).

This idea is also set out by the Italian Ministry of Health in a 2012 publication. In the context of a nodal local health network organised by level of care, some general hospitals could host ECICUs (“extended NICUs”) to which critically ill patients could be referred, sharing the healthcare protocols with their local PICU. This organizational model was also confirmed in the last Italian Permanent Conference for paediatric healthcare guidelines in 2017, where it was affirmed that “If the [critically ill infant] cannot be transferred [to a PICU], he/she must in any case be guaranteed admission, within the time strictly necessary to effect a secondary transfer, to an area dedicated to infants aged under one year in a neonatal ICU and/or general ICU, with his/her clinical management integrated and shared with the specialists at the reference paediatric ICU.” Like its predecessor, this document thus affirms that the management of critically ill infants in general hospitals is the responsibility of neonatologists, in collaboration with the local PICU. This organizational model obviously leaves the management of young children with complex diseases requiring specialist care (neonatal/paediatric surgery, heart surgery, neurosurgery, severe burns, organ transplants, etc.) to the specialised paediatric hospitals, within a regional nodal network classified by level of care [12].

It has also to be considered that lately, several non-invasive techniques have taken hold in the management of critically ill infants, with the consequence for medical and nursing staff to encounter difficulties to maintain proper competence and skills on the invasive procedures [13]. Nevertheless, we believe that the centralization of paediatric intensive care to a handful of qualified and suitably equipped hubs (“extended NICUs” or tertiary PICUs) would enable their staff to gain and maintain the necessary technical and non-technical skills and abilities for the appropriate management of critically ill patients.

## European experiences of “extended NICUs”

A number of NICUs in Italy and Europe have acted as “extended NICUs” in recent years. In Campania, the San Pio hospital in Benevento has managed the intensive care of infants and toddlers since 2004, due to the scarcity of local paediatric intensive care beds [14]. Given its proximity to neighbouring regions with no paediatric intensive care beds at all, it also accepts patients from outside Campania. It treats medical conditions, while patients requiring advanced specialist care are transferred to the regional PICU. On average, it admits around 40 children a year, with many patients discharged through a high intensity home-care process.

In eastern Denmark, the intensive care of infants aged between 0 and 1 years has been centralized to a NICU in Rigshospitalet, the University Hospital in Copenhagen, serving around 2.5 million inhabitants [15]. In 9 years of activity, 35% of patients admitted to the PICU came from other hospitals, and 61% from the same hospital or from home. On average, 100 infants a year were admitted. Of these, 35% were admitted for respiratory diseases, 43% for surgical conditions or postoperative observation, and 18% for other diseases. Mortality was 6.7%, with no difference from the calculated risk for the hospitalized population.

In some European countries with a very similar number of inhabitants to Italy, such as Germany, France, and Spain, paediatric intensive care is provided by NICUs for neonates and by PICUs for paediatric patients (*personal communication by D. De Luca, President of the European Society of Paediatric and Neonatal Intensive Care [ESPNIC]*). This organizational model is flanked by “extended NICUs,” which cover the need for infant and toddler intensive care beds in areas where PICUs are scarce. In countries such as the Netherlands, Greece, and Switzerland that have a much lower population than Italy, paediatric intensive care is organized differently, with a strict division between departments providing neonatal and paediatric intensive care (Table 1).

**Table 1:** Organization of early childhood intensive care in Europe

	NICU (%)	PICU (%)	Extended NICU (%)	NICU (all)/PICU (estimated values — no. of units)	Inhabitants year 2019 (millions)
Germany	40	10	50	135/15	82.9
France	10	15	75	120/20	67.2
Spain	50	25	25	80/28	46.7
Italy	29	16	55	118/23	60.4
Holland	50	50	0	--	17.2
Greece	50	50	0	--	10.7
Switzerland	50	50	0	--	8.5

No data are available comparing the outcomes between countries adopting the PICU or the “extended NICU” models. Hopefully, the ESPNIC or other scientific societies involved in the field will promote in the near future research programs to overcome this issue.

## Transfer of infants and toddlers

The transfer of infants or toddlers from “spoke” centres to the hubs (“extended NICUs” or PICUs) has still not been completely standardized and is often regulated by regional or local protocols. In Italy, a recent survey published by the Neonatal Transfer Study Group of ISN [16] reveals an even distribution of emergency neonatal transfer services in Italy, with 53 stations. Of these, 35 also transfer infants, and one also transfers toddlers. Most infants are transferred for respiratory diseases. Ideally, at least in Italy, the neonatal transfer service should be expanded to infants and small toddlers, with suitably trained dedicated staff. In another transfer organization model, the hubs use retrieval teams for transfers from the “spoke” centres.

Dedicated and specialized transport teams are associated with increased survival and fewer near missed events, such as accidental extubation, cardiovascular arrest, or loss of intravenous lines [17]. In our view, to improve and maintain the technical skills, neonatologists belonging to the neonatal transport team or to the “extended NICUs” should have dedicated time within the working schedule to practice in a PICU settings with higher patient turnover.

## Organization and structuring of “extended NICUs”

The minimum organizational standards for “extended NICUs” and whether they differ from those needed for the intensive care of neonates and preterm infants have been a matter of debate for some time. Ideally, “extended NICUs” should be structured after establishing the organization of the medical and nursing teams, resources, and technologies required for the care of critically ill infants and toddlers, which often differ from those needed for neonates and preterm infants.

“Extended NICUs” should be organized along a family-centred care model, paying attention to the prevention of communicable and contagious diseases, especially in light of the ongoing SARS-COV2 pandemic. They should also contain dedicated equipment for paediatric patients such as invasive and non-invasive ventilation systems and central vascular access, hemodynamic monitoring, and enteral and parenteral nutrition devices [18].

An increase in the number of medical and nursing staff appropriately trained in intensive paediatric medicine is also mandatory. Anaesthesiologists with a dedicated training in neonatal and paediatric critical care could be considered in this setting, since it will reinforce multidisciplinary team work and it will help to overcome the critical paucity of paediatricians that are trained for neonatal and paediatric intensive care in Italy.

## Sub-intensive paediatric units

The sub-intensive paediatric units are set up to take care to infants and children that need accurate monitoring and non-invasive respiratory support, but not intensive care management. They should provide a step-up and step-down admission of children from PICU, thus allowing a faster patient turnover in the PICU and a more efficient management of paediatric critical conditions. Although the efficiency of this model has been recently debated [19], we think that it could be useful in our setting, especially where there is a critical lack of PICU beds and in the presence of an “extended NICU” that could act as a back-up unit in case of clinical deterioration.

The sub-intensive unit could be of special help also in this period of COVID19 pandemic, when exacerbations of viral infection and respiratory failure are expected after a long period of social restrictions [20]. More data are needed to confirm the effectiveness of this health care model.

## Education and training of neonatologists in paediatric critical care medicine

Modern neonatal care shares many aspects with intensive paediatric medicine, but there are also various clinical aspects in the management of critically ill children that are unique to the sector (Table 2) [21]. This means that neonatologists must gain sufficient technical and non-technical knowledge and skills in paediatric critical care medicine (PCCM) to enable the safe management of critically ill infants and toddlers in line with appropriate standards.

**Table 2:** Clinical aspects of NICU/PICU settings

Examples of common issues in neonatal and paediatric intensive care 1. Invasive and non-invasive mechanical ventilation 2. Central vascular access 3. Hemodynamic monitoring 4. Management of severe sepsis and septic shock 5. Congenital heart diseases 6. Pain prevention and management 7. Clinical risk management 8. Prevention and treatment of nosocomial infections 9. Enteral and parenteral nutrition 10. Ethical issues related to end-of-life management 11. Open ICU for the family and family-centred care 12. Family presence during resuscitation manoeuvres or invasive procedure 13. Extracorporeal life support 14. Transport of the critically ill patient
Examples of issues unique to neonatal intensive care 1. Delivery room neonatal resuscitation manoeuvres 2. Life-threatening congenital malformations 3. Bronchopulmonary dysplasia 4. Necrotizing enterocolitis 5. Surfactant deficiency in respiratory distress syndrome 6. Viability of the extremely preterm infant 7. Patent ductus arteriosus
Examples of issues unique to paediatric intensive care 1. Multiple trauma 2. Burns 3. Brain death and organ donation 4. Invasive cardiovascular monitoring (e.g. Swan-Ganz catheter) 5. Intracranial pressure monitoring 6. Meningococcal disease 7. Oncologic diseases 8. Solid organ transplantation−bone marrow transplantation 9. Child abuse

Adapted from Biban P et al. The Journal of Maternal-Fetal and Neonatal Medicine 2011 [21]

There are different specific training paths for neonatologists who want to acquire such skills. The first level of postgraduate training for doctors is specialization. In our opinion, all professionals who manage critically ill infants and toddlers should be paediatric specialists who have acquired suitable skills and undergone appropriate training in neonatal and paediatric intensive care.

Unfortunately, in Italy, there is no established pathway for the study of paediatric intensive medicine when specializing in paediatrics. After graduating in medicine, doctors can specialize either in paediatrics, potentially focusing on neonatology, or in anaesthesiology and intensive care (of adults). We think it could be useful to standardize the paediatric training by adding another one year of paediatric critical care training in dedicated teaching hospital, where PICU is available as subspecialty, for those paediatricians who are willing to work in a NICU/PICU setting. Alternatively, an exchange-residency in ICU could be considered. Conversely, the anaesthesiology — intensive care residency should dedicate 1 or 2 years of rotation in NICUs/PICUs located in teaching hospital, taking care under supervision to critically ill children. Other European countries, including Greece, Norway, and France, include specific neonatal and paediatric intensive medicine training pathways in their paediatric residencies. However, as in all European countries, PCCM falls under the responsibility of paediatricians with specific training in the care of critically ill infants and toddlers.

Another postgraduate training pathway is a Master’s Degree in paediatric intensive care, offered by few Italian universities, that is a 1-year course with an internship in a dedicated PICU.

Scientific societies could undertake to offer certificated and standardized courses for specific paediatric critical care skills such as central vein cannulation, invasive and non-invasive ventilation, management of emergencies or procedural analgesia, and sedation. Some of these already have certified pathways in Europe, such as the European Paediatric Advanced Life Support (EPALS) offered by the European Resuscitation Council (ERC).

To maintain their new skills, medical and nursing staff should train daily through in situ simulations, i.e. in the hospital departments in which they work [22].

ISN-ECICU Study Group has also designed and organized a specific training course on the management of critically ill infants in NICUs. This 2-day course involves four main hands-on sessions. The first session aims to provide basic and advanced ABC resuscitation skills for critically ill children. The second teaches how to manage lung failure with invasive and non-invasive ventilation strategies. The third provides guidelines for the haemodynamic support of paediatric septic shock, including a practical session on ultrasound-guided central venous catheterization and radial arterial cannulation techniques. Finally, the last session includes high-fidelity simulation scenarios to practice non-technical skills. All course teachers are neonatologists with practical experience in PCCM [23].

## Conclusions

Our proposal for healthcare organization is that “extended NICUs” would be set up in general hospitals, in regions or user bases where there is currently a scarcity of PICU beds. These “extended NICUs” would be dedicated to the care of critically ill infants and toddlers, while children with complex conditions requiring specialist care (neonatal/paediatric surgery, heart surgery, neurosurgery, severe burns, organ transplants, etc.) would be managed by the specialist paediatric hospitals, within a regional nodal network classified by level of care. This new model thus affirms that in areas where there is a scarcity of paediatric intensive care beds, the management of critically ill infants and toddlers is the responsibility of neonatologists, in collaboration with the local PICU. Some “extended NICUs” in both Italy and Europe have delivered high-quality healthcare in recent years. However, to enable the delivery of consistently high-quality healthcare, it is essential to establish national or European standards for the facility, technologies, and medical and nursing staff for these “extended NICUs”. In any case, neonatologists must acquire specific technical and non-technical skills and knowledge in intensive paediatric medicine through ad hoc postgraduate training programmes, whether academic or vocational. Scientific societies such as ISN, ESPNIC, and UENPS should undertake the training of all neonatologists and neonatology health workers involved in the management of critically ill infants and toddlers in NICUs and establish the organizational and structural criteria for “extended NICUs”, thus enabling the consistent quality of healthcare offered in Italy and Europe. Finally, we need of further research on monitoring of epidemiological data and clinical outcomes related to the care of severely ill children in Italy and Europe too.

## Abbreviations

ECICUs: Early childhood intensive care units
ESPNIC: European Society of Paediatric and Neonatal Intensive Care
ISN: Italian Society of Neonatology
NICUs: Neonatal intensive care units
PCCM: Paediatric critical care medicine
PICUs: Paediatric intensive care units
UENPS: Union of European Neonatal and Perinatal Societies
